# Do Lignin Nanoparticles Pave the Way for a Sustainable Nanocircular Economy? Biostimulant Effect of Nanoscaled Lignin in Tomato Plants

**DOI:** 10.3390/plants13131839

**Published:** 2024-07-04

**Authors:** Ciro Tolisano, Dario Priolo, Monica Brienza, Debora Puglia, Daniele Del Buono

**Affiliations:** 1 Dipartimento di Scienze Agrarie, Alimentari e Ambientali, Università degli Studi di Perugia, Borgo XX Giugno 74, 06121 Perugia, Italy; ciro.tolisano@dottorandi.unipg.it (C.T.); dario.priolo@unipg.it (D.P.); 2 Dipartimento di Scienze, Università degli Studi della Basilicata, Via dell’Ateneo Lucano 10, 85100 Potenza, Italy; monica.brienza@unibas.it; 3 Department of Civil and Environmental Engineering, University of Perugia, Strada di Pentima 5, 05100 Terni, Italy

**Keywords:** lignin, nanomaterials, waste valorization, *Lycopersicon esculentum*, biostimulant

## Abstract

Agriculture has a significant environmental impact and is simultaneously called to major challenges, such as responding to the need to develop more sustainable cropping systems with higher productivity. In this context, the present study aimed to obtain lignin nanoparticles (LNs) from pomace, a waste product of the olive oil chain, to be used as a nanobiostimulant in tomato plants. The biostimulant effect of this biopolymer is known, but its reduction to nanometer size can emphasize this property. Tomato plants were subjected to different LN dosages (25, 50, and 100 mg L^−1^) by foliar application, and inductive effects on photosynthetic machinery, aerial and root biomass production, and root morphology were observed. The treated plants showed increased efficiency in catching and using light, while they reduced the fraction dissipated as heat or potentially toxic to cells for the possibility of creating reactive oxygen species (ROS). Finally, this benefit was matched by increased pigment content and a stimulatory action on the content of nitrogen (NBI) and antioxidant substances such as flavonoids. In conclusion, the present study broadens the horizon of substances with biostimulant action by demonstrating the validity and efficacy of nanobiostimulants obtained from biological residues from the olive oil production chain.

## 1. Introduction

In the coming years, agriculture will be called upon to meet the growing demand for food due to an ever-increasing world population in the context of climate change [1,2]. On the other hand, the environmental impact of agriculture is no longer sustainable, as this activity disperses pollutants, releases greenhouse gases, and creates considerable quantities of waste that are challenging to manage [3].

One of the main weaknesses of the modern agricultural system is the general adoption of a production linear model (linear economy) that “takes, makes, uses and disposes of” nonrenewable natural resources [4]. This leads to the need to implement effective strategies in line with the principles of the circular economy that see waste from the agro-industrial supply chain, for example, as a potential resource for obtaining different types of materials and compounds [5]. For this, the circular economy model creates a new regenerative paradigm that places centrality on valorizing limited resources, intending to ensure global prosperity by containing waste generation as much as possible or by zeroing it out. This is possible because waste from consumed biological resources falls entirely into a virtuous cycle as it is recovered and reused [6].

The circular economy increasingly involves nanostructured materials derived from waste biomass [2,7]. Nanomaterials represent a convenient opportunity to integrate and enhance the circular economy by creating an essential connection with agricultural research because of their exceptional properties [5]. In this view, reducing the environmental impact of agriculture and increasing its productivity through a shift to a multifunctional biorefinery model are increasingly addressing the use of waste to obtain functional biobased nanostructured products. In particular, some nanostructured materials can stimulate benefits in crops by acting as plant growth promoters [8,9]. For this reason, today, the implementation of nanotechnology in agriculture is the subject of intense research and can also have considerable synergies with the circular economy.

A convenient strategy that enhances the value of waste while reducing its environmental impact is the development of biostimulants, which are innovative materials capable of increasing crop performance under normal conditions and environmental stress [6]. Recent scientific evidence includes some nanostructured materials among plausible candidates for developing new biostimulants, the so-called nanobiostimulants [1]. Briefly, biostimulants are materials of natural origin that can be derived from a wide range of raw biomasses. For simplicity, they have been grouped into biostimulants of microbial and nonmicrobial origin. The latter include protein hydrolysates, plant and algae extracts, humic substances, certain organic biopolymers, and inorganic compounds [3,10].

In this respect, the olive oil supply chain is very interesting as it produces large amounts of waste that contain a plethora of substances worthy of attention and that, if recovered, can represent interesting bioactives [11]. They can also be the basis for developing innovative materials, including those that are nanostructured [2]. Olive oil production that relies on two- or three-phase extraction technologies produces solid or liquid wastes such as wastewater and pomace [12]. Due to its chemical composition, pomace has a robust environmental impact [13,14]. However, such a matrix has several components, which, if recovered, can find numerous implementations from a technological and commercial point of view. Olive pomace has, among other things, a very high content of organic matter, consisting mainly of fiber (~60%) such as lignin (30–40%), cellulose (up to 30%), and hemicellulose (about 8%), along with significant amounts of protein (up to 10%), polysaccharides (up to 7%), fatty acids, and phenols and polyphenols [14,15].

Among all the substances in olive pomace, lignin is considered a biopolymer that deserves attention as it can be used in various applications to obtain bioplastics, biofuels, nanoparticles, and nanocomposites [16,17,18]. Recent scientific evidence has also shown that lignin can find a prominent space to develop products to be used in agriculture. In fact, this biopolymer, because of its chemical composition, can positively influence the growth and development of certain crops and positively affect the soil and microbial communities [19]. Indeed, raw lignin and its derivatives can induce and promote the growth of some crops, stimulating the germination and early seedling stages and improving certain physiological, morphological, and biochemical traits in adult plants [20,21]. These effects have generally been attributed to the ability of lignin to exert a hormone-like action [22].

Furthermore, in the context of nanoscaled materials, it is noteworthy to note the recently ascertained effect of lignin nanoparticles (LNs) on promoting certain benefits in maize plants [9,23]. Some studies showed that LNs conspicuously improved plant biomass, pigment content, and cellular redox state [2,23,24]. Furthermore, lignin nanoparticles stimulated tomato seeds for their capacity to pass the seed tegument and penetrate tissues [25]. Nonetheless, the recovery of lignin from waste biomass and its conversion to nanomaterials to be used as biostimulants in agriculture is a nascent and challenging area of research, despite its undoubted potential to show bioactivity in crops.

In light of the above, the present study applied a previously published procedure to pomace [2], based on an extraction technology involving an ionic liquid (IL) consisting of triethylamine and sulfuric acid ([TEA][HSO_4_]) to recover and nanoscale lignin [2]. It has been observed that ILs [TEA][HSO_4_] and imidazolium hydrogen sulfate effectively cleave β-O-4, the most important bonds in all types of lignin, promoting a high depolymerization rate and facilitating lignin precipitation [26,27]. Then, the effect of the LN thus obtained was tested in tomato plants cultivated in a growth chamber by investigating the effects on aerial and root biomass, root phenotyping, photosynthetic machinery, and some biochemical traits. Therefore, the objectives of this study were multiple since it aimed at the valorization of waste biomass by obtaining nanostructured lignin to be used as a biostimulant for a horticultural crop of pivotal importance on a global scale. Finally, this study aimed to obtain any evidence or indication about how the treatment could promote any positive effect.

## 2. Results

### 2.1. LN Characterization

**Figure 1:**
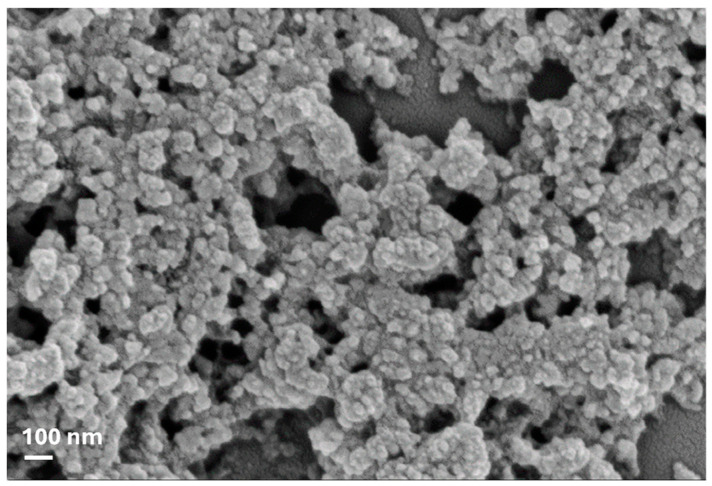
FESEM image of LNs obtained from olive pomace.

FTIR characterization of pure lignin nanoparticles was confirmed, according to an already previously published paper [2]. The presence of phenolic O-H and aliphatic C-H in methyl groups and the stretching of -C=O indicated that guaiacyl (G) and syringyl (S) groups were present, while the strong vibrations of C-C, C-O, and C=O bond stretching indicated that the condensed G was more representative than etherified G. Analysis of thermal behavior also showed that lignin decomposes in three distinct stages with the formation of low-molecular-weight products in the entire range from 150 to 800 °C (data already published), as already reported by Tolisano et al. [2]. Therefore, the biomass pretreatment confirmed the efficiency of IL in controlling lignin particle size at the nanoscale (Figure 1).

### 2.2. Effect of LNs on Some Different Aspects of Tomato Photosynthesis

**Figure 2:**
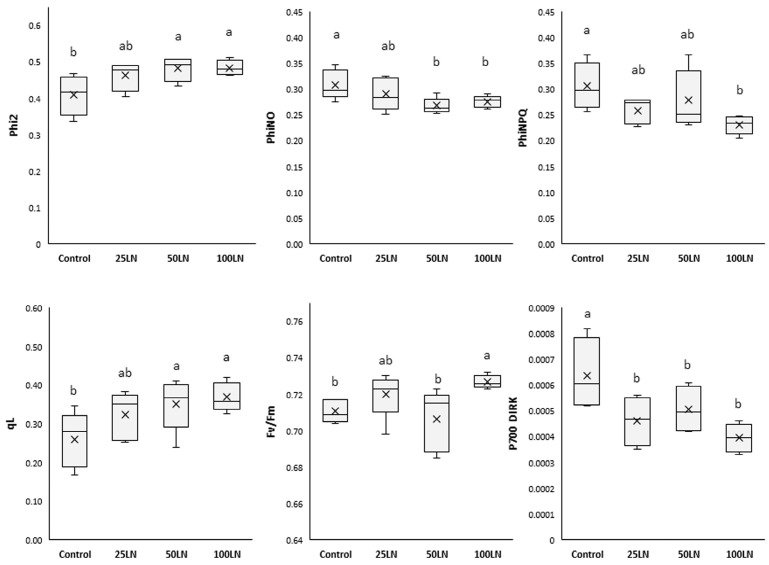
Effects of LNs on the photosynthetic activity of tomato plants. The following parameters are reported: Phi2 (the efficiency of PSII), PhiNO (the non-regulated dissipation of light energy), PhiNPQ (the photo-protective non-photochemical quenching), qL (the open state of PSII), Fv/Fm (the photochemical efficiency of PSII), and P700 DIRK (the dark-interval relaxation kinetics of P700). Different letters indicate that values are significantly different according to Duncan’s multiple comparison test (*p* < 0.05).

LN applications influenced the photosynthetic activity of tomato plants, as indicated by the parameters investigated (Figure 2). In detail, regarding the functionality and activity of photosystem II, the quantum yield (Phi2) increased significantly, compared to control samples, in plants treated with LN50 and LN100, while those treated with LN25 did not show substantial differences with the other treatments.

The fraction of light lost via non-regulated processes (PhiNO) decreased significantly in samples treated with LN50 and LN100 compared to control samples. Differently, LN25 did not affect this parameter. As for the fraction of light dispersed by plants as non-photochemical quenching (PhiNPQ), it was found that LN100 lowered this parameter while remaining unaffected by the other two dosages. Regarding the PSII centers resulting in an open state (qL), LN50 and LN100 significantly increased it, which, differently, remained unaffected by the lowest concentration, LN25. As for the maximal quantum efficiency of PSII (Fv/Fm), this parameter significantly increased in samples treated with LN100 compared to control samples. The other two treatments did not change Fv/Fm in tomato plants. Additionally, all treatments decreased the dark-interval relaxation kinetics of P700 (P700 DIRK) compared to controls.

**Figure 3:**
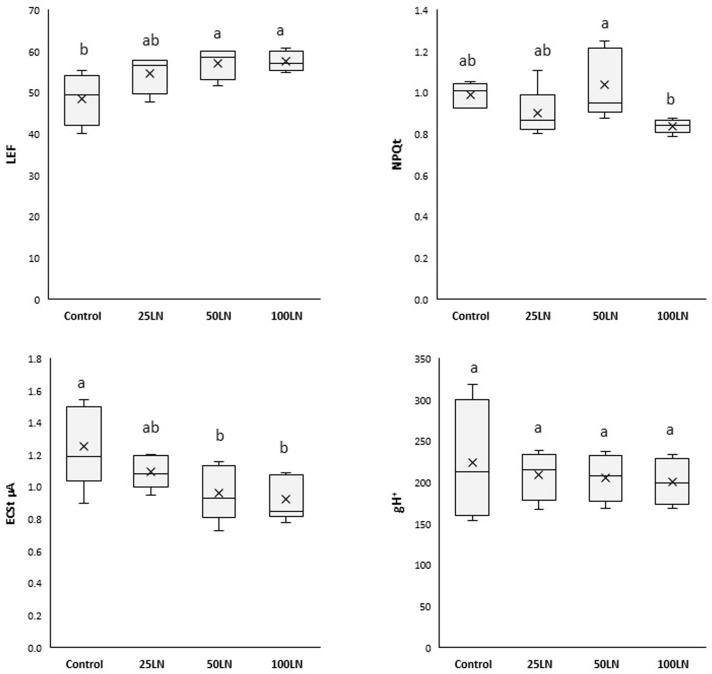
Effects of LN treatments on the photosynthetic activity of tomato plants. LEF is the linear electron flow, NPQt is the total non-photochemical quenching, ECSt is the total electrochromic shift, and gH^+^ is the proton conductivity of the thylakoid membrane. Different letters indicate that values are significantly different according to Duncan’s multiple comparison test (*p* < 0.05).

Plants treated with LN50 and LN100 showed a consistently higher linear electron flow (LEF), while LN25 did not affect tomato plants (Figure 3). A decrease in the total electrochromic shift (ECSt) was recorded for samples treated with LN50 and LN100, while no differences were observed for LN25 compared to control samples. Finally, the total non-photochemical quenching (NPQt) and the proton conductivity of the thylakoid membrane (gH^+^) were generally unaffected by any LN treatment.

### 2.3. Effect of LN Treatments on Plant Growth at Shoot and Root Level

LN foliar applications significantly affected tomato plant growth, not only at the shoot level but also at the root level. In particular, regarding the former, all the LN treatments determined significant leaf thickness increases, resulting in higher values than those found in control samples (Table 1). Moreover, LN100 also prompted increases in the shoot fresh weight. In addition, plants treated with LN25, LN50, and LN100 showed a higher shoot dry weight than the control group. Finally, as for the shoot height and number of leaves, these growth parameters were generally unaffected by any treatments applied to tomato plants.

Going forward with root phenotyping, total root length increased with LN applications in a dose-dependent manner (Table 2). The number of root tips was higher than the control for all treatments, regardless of the dosage applied. In addition, in the case of LN100, the surface area was significantly increased. Moreover, regardless of the concentration applied to the tomato, all plants treated with LNs showed a higher root fresh weight than control samples.

LN100 treatment stimulated root biomass production, as evidenced by the recorded values of root dry weight. In contrast, LN25 and LN50 did not prompt changes in this parameter compared to control samples. Finally, all LN dosages applied did not affect the root average diameter or volume.

### 2.4. Biochemical Determinations (TPC, TFC, Soluble Carbohydrates, SPAD, NBI)

The SPAD index, representing the chlorophyll content, increased for all LN concentrations compared to the control samples (Table 3). As for the nitrogen balance index (NBI), plants treated with LN100 showed higher values than untreated plants, while the other dosages did not affect this parameter. Total phenol content (TPC) increased in the LN25 group, while the other LN treatments showed no significant differences compared to the control samples. Differently, the total flavonoid content (TFC) was enhanced by all LN treatments, regardless of the dosage applied. Finally, the soluble carbohydrate concentration was unaffected by all the LN treatments.

## 3. Discussion

Developing new and eco-friendly nanomaterials acting as nano-growth promoters can play a crucial and pioneering role in supporting and improving agriculture by reducing the use of or replacing conventional products characterized by high environmental impact, such as synthetic chemical fertilizers, pesticides, etc. [28]. In addition, if such materials are obtained from waste substances, the technological solution takes on a strategic value that fully aligns with the founding concept of the circular economy.

In the present research, in line with the above, olive pomace, a substance commonly disposed of as waste or used by combustion for energy purposes, was used as a source of a potential biostimulant based on lignin nanoparticles. As already reported by Tolisano et al. [2], the procedure to process biomass based on the use of the ionic liquid [TEA][HSO_4_] was efficient in controlling the size of lignin particles, permitting the obtaining of nanoparticles with average diameter values of 40 ± 3 nm (Figure 1) and allowing for the intact maintenance of the lignin functional groups, thus highlighting the excellent character of ILs for obtaining lignin from waste biomass [29].

Regarding tomato plants, the results of this study showed that LNs stimulated numerous specific aspects of the photosynthetic machinery, generally following a dose-response effect (Figure 2 and Figure 3). This effect is significant because the photosynthetic machinery carries out a fundamental anabolic process that converts solar energy into chemical energy (carbohydrates), thus strongly affecting crop yields [30]. Improving the efficiency of this process is, therefore, fundamental for increasing crop productivity in general and that of tomato samples in the specific study.

Our results showed that LN treatments at the highest dosages strongly influenced both photosystems (PSII and PSI) (Figure 2). In fact, plants treated with 50 and 100 mg L^−1^ LNs (LN50 and LN100) showed general improvements in the studied parameters. In particular, the effect of the LNs resulted in an improved ability of PSII and PSI to transmit light energy to each other, as indicated by Phi2 (the amount of light absorbed by PSII for photosynthetic purposes) and P700 DIRK (the relaxation rate of PSI). In particular, the decrease in the latter parameter indicates an improved readiness of the PSI to proceed with the transport of energy derived from photons transmitted by the PSII [31]. In addition, it is noteworthy that for the treatments mentioned above, the data of increased efficiency of PSII were corroborated by a significant decrease in the amount of light non-photochemically used and mainly dissipated as heat (PhiNPQ, non-photochemical quenching), as well as that translated into toxic forms to the cell (PhiNO, non-regulatory energy dissipation), which can be progenitor of highly oxidizing substances such as reactive oxygen species (ROS) [32]. Finally, the LNs also stimulated an increase in the active centers of PSII (qL). Despite this, such an effect did not result in an increased energy flux that could potentially damage the photosystems. Indeed, as indicated by the Fv/Fm that represents the health status of PSII, the LN improved the capacity of tomato plants to carry out photosynthesis. In addition, the highest LN dosage also improved the Fv/Fm value, thus indicating an ameliorated state of PSII [33].

Due to the improved efficiency of the two PSs, the LN-treated plants showed a higher linear electron flux (LEF) than the control samples (Figure 3). In other words, the LNs increased the efficiency of plants in extracting electrons from H_2_O and transferring them through the PSII to the PSI, thus finalizing a higher production of NADPH. In this way, it should be considered that photosynthesis is a highly regulated and coordinated electron flow associated with the translocation of protons from the stroma to the lumen [34], thus determining the formation of an electrochemical gradient exploited by ATP synthase for synthesizing chemical energy in ATP [35]. The decrease in electrochromic shift (ECSt) recorded for LN-treated samples (Figure 3) indicates that these nanoparticles stimulated ATP production. In particular, the amplitude of the ECSt is proportional to the proton motive force (*pmf*), and its decreased levels indicate an increase in ATP synthesis through the catalytic action of ATPase [34]. The explanation for such an overall effect of nanoparticles on the photosynthetic apparatus has been proposed by pioneering studies and hypotheses that showed that some nanostructured nanomaterials, because of their electronic properties, can support photosynthesis [1]. Nanomaterials can increase the ability of the crop to harvest and use light, thus producing more biomass. Indeed, it has been shown that, in some cases, nanoparticles and carbon-based nanomaterials can function as an additional light-harvesting system, intercepting the fraction of unused light in the photosynthetic spectrum by plants or acting as a photo reflector and, thus, transferring radiant energy to reaction centers [36,37,38,39]. Moreover, it has been shown that such effects result from the electronic properties of the materials and mainly occur in those containing aromatic and polyaromatic structures and domains of carbons with sp^2^ hybridization [40]. From a molecular point of view, lignin has these characteristics in terms of its structure, bonding, and electronic organization [23].

Regarding aerial biomass production, the data show a clear beneficial effect generally promoted by the different LN treatments in terms of leaf thickness and fresh and dry weight, while the other treatments investigated were unaffected (Table 1). In addition, the root phenotyping (Table 2) showed an inductive effect on some root parameters in response to lignin nanoparticle treatment. Specifically, LNs stimulated root length and the total number of tips, and for the highest dosage of LN100, fresh and dry weights of roots were also significantly increased.

In general, nanomaterials can positively affect aerial biomass production, and this action can mainly depend on their chemical composition, shape, and size [7,9]. In addition, in the case of nanoscaled lignin, it is necessary to emphasize its bio- and eco-friendly character and its beneficial effects on structure, chemical composition, and soil fertility [41]. Moreover, particular emphasis should be placed on the beneficial effect we found on the root system, as it is well known that improving this trait results in better acquisition of nutrients from the soil. To explain these inductive effects, we can refer to the already-discussed promotion of photosynthetic machinery by nanoscaled materials. However, lignin and, of course, nanoscaled lignin, due to its chemical composition mainly based on three phenylpropane units (guaiacyl—G, syringyl—S, and p-hydroxyphenyl), can also promote the development of plant biomass thanks to other structural properties [23]. In particular, these monolignols are phenolics that can exert hormone-like action on treated crops by promoting their development [9]. For instance, it has been shown that in plants, phenylpropanoid compounds stimulate L-tryptophan biosynthesis and indole-3-acetic acid (IAA) transformation [42]. Such an effect results in the stimulation of plant development and biomass production. In addition, the syringyl group can exert a gibberellin-like action by inducing the development of the treated plants [43,44]. The other relevant component that confers bioactivity to lignin is the guaiacyl group, which can positively affect the development of the crop aerial and root parts [44,45]. The enhanced biomass production can also be related to the presence of the p-hydroxyphenyl group in this complex biomolecule [42]. More generally, all the above results align with the recent bibliography, which shows that lignin has a multifunctional effect and can stimulate photosynthesis, activate rubisco, induce photosynthesis-related proteins and regulatory proteins, and have a hormonal-like effect [46].

Successively, some biochemical parameters were investigated to ascertain the eventual other effects prompted by LNs in tomato plants. In particular, the content of chlorophylls (indicated as the SPAD index), the nitrogen balance index (NBI), and the content of total phenols, flavonoids, and soluble carbohydrates were investigated (Table 3). As for the photosynthetic pigment, LNs generally increased its content regardless of the dosage applied [1,36]. Chlorophyll is a pivotal pigment that plays a determinant role in photosynthesis as it can absorb light in the visible region and use it in the reaction centers to support this anabolic chemical energy production process [47]. Some nanomaterials can improve photosynthetic machinery, as demonstrated in this study (Figure 1 and Figure 2), and stimulate chlorophyll content. In turn, this enables the light-harvesting systems of chloroplasts to absorb and convert light more effectively [48]. Furthermore, it has been shown that phenols and monolignols present in lignin can contribute to increasing the content of chlorophylls; this is related to an improved plant capacity to uptake nutrients in general and nitrogen in particular [49]. This result for the highest LN dosage aligns with our results, as LN100 stimulated a substantial increase in the nitrogen balance index (NBI), which is indicative of the nitrogen nutritional status of plants.

Our experiments evidenced that LN promoted significant increases in the content of flavonoids, while phenols remained generally unchanged, except for the lowest dosage. These two classes of metabolites cover some essential functions in plants. In particular, flavonoids are involved in molecular signaling in cells that operate in plant protection from biotic and abiotic stresses and plant acclimation [50]. In addition, they are attracting interest for their protective role in human health and their benefits in terms of their antioxidant, antibacterial, and thrombolytic activity [51]. Therefore, the increase in these compounds prompted by lignin nanoparticles is noticeable. Finally, we investigated the content of soluble carbohydrates that could indicate eventual stress prompted by the treatments since they are involved in osmoprotective adjustments to deal with toxic agents or stressors [52]. Our results evidenced that LNs did not modify their content, thus indicating that the treatments did not result in significant stress based on the above parameters.

## 4. Materials and Methods

### 4.1. Pomace Treatment to Obtain LNs

An ionic liquid consisting of [Et_3_NH][HSO4], synthesized according to Cequier et al. [53], was used to treat the pomace product. The treatment to obtain nanoparticle lignin was then conducted using the aforementioned IL, mixing the dry pomace with it (1:10, *w*:*w*), and adding 5 wt% of water. After a brief contact, additional H_2_SO_4_ was added to the mixture to maximize the lignin extraction yield. Details of the extraction procedures are given in Tolisano et al. [2]. The LNs were then characterized chemically (FT-IR, Jasco FT-IR 615 spectrometer (Jasco Corporation, Tokyo, Japan), range of 4000–600 cm^−1^, transmission mode), morphologically (FESEM, Supra 25-Zeiss, 5 kV—Zeiss, Oberkochen, Germany), and for their thermal stability (by heating the samples from 30 to 900 °C at 10 °C min^−1^ under nitrogen flow (250 mL min^−1^) using a Seiko Exstar 6300). The results were the same as those already reported in the previously published paper [2], confirming the validity and reproducibility of the method applied to obtain LNs. For simplicity, here we report an FE-SEM image (Figure 1).

### 4.2. Tomato Growth Conditions, LN Treatments, Physiological and Morphological Determinations, and Photosynthetic Activity

The cultivar Rio Grande (*Lycopersicon esculentum*) was used for the experiments. The seeds were sown individually in plastic pots containing commercial peat, and after 5 days of germination in the dark, the pots were placed in a growth chamber under a photoperiod of 12/12 h (day/night), with a light intensity of 300 μmol m^−2^ s^−1^, at a constant temperature of 24 °C. Growing seedlings were irrigated daily with water until they reached a 75% field moisture capacity.

Tomato seedlings were treated with LNs by two foliar spray applications at 4 and 5 weeks after sowing, with solutions containing 0 (control), 25, 50, or 100 mg L^−1^ LN. For each treatment, 5 replicates were carried out according to a completely randomized experimental design. According to a previous study [2], the above concentrations were chosen because foliar treatments on plants with nanoparticles at concentrations higher than 100 mg L^−1^ can result in some phytotoxic effects.

Six-week-old plants were harvested for the physiological, morphological, and biochemical determinations as indicated below. The spectroscopic analyses were conducted to determine the photosynthetic activity and some aspects of the photosynthetic machinery following the treatments on intact and fully expanded leaves using the MultispeQ device (PHOTOSYNQ INC., East Lansing, MI, USA) software Photosynthesis RIDES 2.0. Data were collected and analyzed on the web platform PhotosynQ (http://www.photosynq.org) [54]. The measurements were taken on all replicates of each experimental group. In particular, the following parameters were studied: the quantum yield of PSII (Phi2), the fraction of light that can be lost via non-regulated processes (PhiNO) or released as non-photochemical quenching (PhiNPQ), the maximal quantum efficiency of PSII (Fv/Fm), the fraction of PSII centers, which are in the open state (qL), the dark-interval relaxation kinetics of P700 (P700 DIRK), the linear electron flow from the antennae complexes into PSII (LEF), the total non-photochemical quenching (NPQt), the total electrochromic shift (ECSt), and the proton conductivity of the thylakoid membrane (gH^+^).

Regarding biomass production and morphological traits, shoot biomass production and quality were evaluated by measuring shoot height and the number of leaves. Furthermore, the leaf thickness was recorded. Then, root biomass production was investigated, and phenotyping was carried out on the scanned root using RhizoVision Explorer v2.0.3.0, according to Seethepalli et al. [55]. In particular, the total root length (cm), number of root tips, diameter (mm), surface area (cm^2^), and volume (cm^3^) have been evaluated. Finally, the fresh mass of shoots and roots was measured and oven-dried at 60 °C until the samples reached a constant weight (dry weight).

### 4.3. Biochemical Determinations

Among the photosynthetic parameters collected using MultispeQ, the relative chlorophyll content was determined as the SPAD index. Finally, the nitrogen balance index (NBI) was estimated based on non-destructive measurements with the Dualex device (Force A, Orsay, France), and all measurements were performed on the middle of the young, fully developed upper leaf.

Total phenolic content (TPC), total flavonoid content (TFC), and soluble carbohydrates were determined by extracting 0.25 g of fresh leaf samples in 2.5 mL of methanol, and this suspension was then centrifuged (6000 rpm, 20 min). The Folin–Ciocalteu method was adopted for TPC, and phenol content was referred to as gallic acid equivalent (GAE) g^−1^ [56]. TFC was determined spectrometrically, according to Atanassova et al. [57], and was expressed as mg of catechin equivalents (CEs) g^−1^. Soluble carbohydrates were determined by the anthrone method, according to Al Murad and Muneer [58]. From the methanolic extract, 50 µL of supernatant was taken, brought to a final volume of 1 mL with distilled water, and added to 2.5 mL of a 0.2% anthrone reagent. The solutions were heated (100 °C, 10 min), and, after cooling, the absorbance of the samples was measured spectrophotometrically at 620 nm. Glucose was used to construct the calibration curve, and the total soluble carbohydrates were expressed as mg g^−1^ FW.

### 4.4. Statistical Analyses

The collected data were subjected to a statistical analysis through one-way analysis of variance (ANOVA), and significant differences were assayed by Duncan’s test (*p* < 0.05). The experiment was carried out according to a completely randomized design with four treatments (0 mg L^−1^, 25 mg L^−1^, 50 mg L^−1^, and 100 mg L^−1^ LN) and five replicates per treatment. The data presented in the tables represent the mean value ± standard deviation.

## 5. Conclusions

This study has shown that developing and using innovative nanomaterials from agro-industrial waste and their application on tomato plants can promote relevant biological effects and, in turn, open a new avenue for nanobiotechnology to support agriculture. In fact, obtaining nanobiostimulants from waste substances can play an important role in cropping systems from a circular economy perspective, improve the utilization of biological resources, and reduce the environmental impact of cropping systems. Our research has shown that lignin nanoparticles can effectively stimulate the photosynthetic machinery of tomato plants. This effect is noteworthy and deserves attention, as positive conditioning of some aspects of the photosynthetic machinery function can effectively affect the ability of plants to convert solar energy into chemical energy, thus enhancing crop yield. In addition, the data also showed beneficial effects on biomass production and photosynthetic pigments. The latter result is significant because these molecules play a crucial role in photosynthesis, and an increase in their content can better support and improve the anabolic process of photosynthesis. Finally, this study also showed that lignin nanoparticles can promote a significant increase in the content of flavonoids, substances of interest, as they assume a key role in plant protection and are important for human nutrition.

In conclusion, among the prospects for future research, it will undoubtedly be necessary to ascertain the efficacy of lignin nanoparticles in tomato plants until the end of the entire crop cycle. However, the data obtained are encouraging in this study since bioactive substances and biostimulants that bring benefits in the early stages of development retain this trait throughout the crop life cycle.

Another aspect of primary importance lies in carefully evaluating the mechanisms by which nanobiostimulants operate to make this new frontier of biostimulant substances increasingly efficient. Although what we have proposed in this study refers to a natural biopolymer that is widely found in nature, a variety of nanomaterials can play an important role in improving agricultural practices. However, before their use, a holistic approach is needed to understand their benefits and potential negative impacts, in addition to a clear mechanistic understanding of their effects.

## Figures and Tables

**Table 1:** Shoot analyses of tomato plants untreated (Control) and treated with different concentrations of LNs.

Treatment	Shoot Height (cm)	Number of Leaves (n°)	Leaf Thickness (mm)	Fresh Weight per Plant (g)	Dry Weight per Plant (g)
Control	13.8 ± 0.5 a	34 ± 2 a	0.40 ± 0.07 b	6.59 ± 0.74 b	0.84 ± 0.14 c
LN25	13.7 ± 0.5 a	35 ± 3 a	0.73 ± 0.07 a	7.68 ± 0.48 a	1.22 ± 0.04 a
LN50	14.4 ± 0.4 a	36 ± 2 a	0.65 ± 0.13 a	7.35 ± 0.31 ab	1.04 ± 0.04 b
LN100	14.3 ± 0.2 a	36 ± 1 a	0.66 ± 0.25 a	7.54 ± 0.45 a	1.03 ± 0.05 b

Different letters are statistically significant according to Duncan’s multiple comparison test (*p* < 0.05).

**Table 2:** Root analyses of tomato plants untreated (Control) and treated with different concentrations of LNs.

Treatment	Total Length (cm)	Root Tips (n°)	Diameter (mm)	Root Area (cm^2^)	Volume (cm^3^)	Root Fresh Weight (g)	Root Dry Weight (g)
Control	5094.3 ± 792.1 c	810 ± 113 b	0.76 ± 0.05 a	121.7 ± 26.6 b	3.59 ± 1.30 a	1.33 ± 0.10 b	0.25 ± 0.03 b
LN25	5567.7 ± 205.1 bc	1091 ± 19 a	0.76 ± 0.07 a	133.5 ± 10.2 ab	3.91 ± 7.16 a	2.53 ± 0.72 a	0.30 ± 0.05 ab
LN50	6485.1 ± 649.5 ab	1207 ± 233 a	0.73 ± 0.06 a	148.5 ± 4.2 ab	4.19 ± 3.71 a	2.77 ± 0.01 a	0.28 ± 0.02 ab
LN100	6654.9 ± 346.2 a	1113 ± 53 a	0.77 ± 0.04 a	157.7 ± 16.7 a	4.65 ± 8.36 a	2.74 ± 0.17 a	0.32 ± 0.01 a

Different letters are statistically significant according to Duncan’s multiple comparison test (*p* < 0.05).

**Table 3:** Chlorophyll content (SPAD), nitrogen balance index (NBI), total phenol content (TPC), total flavonoid content (TFC), and soluble carbohydrates in tomato plants untreated and treated with different concentrations of LNs.

Treatment	SPAD	NBI	TPC (mg g^−1^ FW GAE)	TFC (mg g^−1^ FW)	Soluble Carbohydrates (mg g^−1^ FW)
Control	32.7 ± 3.8 b	68.04 ± 6.66 b	1.95 ± 0.47 b	1.42 ± 0.14 b	1.22 ± 0.29 a
LN25	36.8 ± 0.8 a	72.46 ± 10.38 ab	2.48 ± 0.34 a	1.78 ± 0.06 a	1.36 ± 0.34 a
LN50	36.4 ± 3.0 a	75.06 ± 4.29 ab	2.27 ± 0.27 ab	1.73 ± 0.06 a	1.16 ± 0.24 a
LN100	37.4 ± 1.6 a	82.26 ± 6.62 a	1.86 ± 0.19 b	1.79 ± 0.06 a	1.30 ± 0.30 a

Different letters are statistically significant according to Duncan’s multiple comparison test (*p* < 0.05).

## Data Availability

Dataset available on request from the authors.
